# Antenatal exercise in overweight and obese women and its effects on offspring and maternal health: design and rationale of the IMPROVE (Improving Maternal and Progeny Obesity Via Exercise) randomised controlled trial

**DOI:** 10.1186/1471-2393-14-148

**Published:** 2014-04-26

**Authors:** Sumudu N Seneviratne, Graham K Parry, Lesley ME McCowan, Alec Ekeroma, Yannan Jiang, Silmara Gusso, Geovana Peres, Raquel O Rodrigues, Susan Craigie, Wayne S Cutfield, Paul L Hofman

**Affiliations:** 1 Liggins Institute, University of Auckland, Auckland, New Zealand; 2 Gravida: National Centre for Growth and Development, Auckland, New Zealand; 3 Department of Obstetrics and Gynaecology, Faculty of Medical and Health Science, University of Auckland, Auckland, New Zealand; 4 Middlemore Hospital, Counties Manukau Distrct Health Board, Manukau, New Zealand; 5 Department of Statistics, University of Auckland, Auckland, New Zealand

**Keywords:** Pregnancy, Overweight, Obese, Antenatal exercise, Offspring, Birth weight, Neonatal body composition, Fetal growth, Pregnancy outcomes

## Abstract

**Background:**

Obesity during pregnancy is associated with adverse outcomes for the offspring and mother. Lifestyle interventions in pregnancy such as antenatal exercise, are proposed to improve both short- and long-term health of mother and child. We hypothesise that regular moderate-intensity exercise during the second half of pregnancy will result in improved maternal and offspring outcomes, including a reduction in birth weight and adiposity in the offspring, which may be protective against obesity in later life.

**Methods/Design:**

The IMPROVE (Improving Maternal and Progeny Risks of Obesity Via Exercise) study is a two-arm parallel randomised controlled clinical trial being conducted in Auckland, New Zealand. Overweight and obese women (BMI ≥25 kg/m2) aged 18–40 years, with a singleton pregnancy of <20 weeks of gestation, from the Auckland region, are eligible for the trial. Exclusion criteria are ongoing smoking or medical contra-indications to antenatal exercise.

Participants are randomised with 1:1 allocation ratio to either intervention or control group, using computer-generated randomisation sequences in variable block sizes, stratified on ethnicity and parity, after completion of baseline assessments. The intervention consists of a 16-week structured home-based moderate-intensity exercise programme utilising stationary cycles and heart rate monitors, commencing at 20 weeks of gestation. The control group do not receive any exercise intervention. Both groups undergo regular fetal ultrasonography and receive standard antenatal care. Due to the nature of the intervention, participants are un-blinded to group assignment during the trial.

The primary outcome is offspring birth weight. Secondary offspring outcomes include fetal and neonatal body composition and anthropometry, neonatal complications and cord blood metabolic markers. Maternal outcomes include weight gain, pregnancy and delivery complications, aerobic fitness, quality of life, metabolic markers and post-partum body composition.

**Discussion:**

The results of this trial will provide valuable insights on the effects of antenatal exercise on health outcomes in overweight and obese mothers and their offspring.

**Trial registration:**

Australian New Zealand Clinical Trials Registry ACTRN12612000932864.

## Background

The number of women entering pregnancy in an overweight or obese state has increased steadily in developed countries over the last three decades [1-4], and is also increasing in developing nations [5]. Maternal overweight and obesity are associated with adverse pregnancy outcomes, including higher risk of gestational diabetes, pre-eclampsia, operative delivery, fetal and perinatal mortality and morbidity, and excessive birth weight [6,7]. Maternal obesity can also result in long-term health problems for the offspring, secondary to perinatal problems and to intrauterine and postnatal programming effects [7,8], including an increased obesity risk in childhood and adulthood [9].

However, it may be possible to alter programming of the offspring of obese women to a healthier phenotype by interventions in pregnancy [10]. There is growing evidence to suggest the prenatal environment may play a role in programming obesity risk [11]. The link between maternal and offspring obesity can be explained by the ‘early-life hyper nutrition’ pathway where exposure to over-nutrition during fetal or early postnatal life may lead to perturbations of adipogenesis and/or appetite control mechanisms, creating a propensity to later obesity [8]. There is evidence from animal models that exposure to maternal obesity *in utero* can lead to offspring adiposity and cardiovascular and metabolic dysfunction, which may result from developmentally programmed hyperphagia, physical inactivity, and altered adipocyte metabolism [12]. Catalano postulates the existence of a vicious cycle of obesity, whereby maternal obesity leads to fetal overgrowth and subsequently post-natal obesity. Overweight/obese children tend to become obese adults, and the females, when pregnant, will increase nutrient supply to the fetus leading in fetal overgrowth in the next generation [13,14]. Further, it is proposed that offspring obesity may be propagated and enhanced in early life because of the abnormal metabolic milieu *in utero* in overweight and obese women [13,14]. This raises the potential of *in utero* therapy to prevent downstream obesity and chronic disease obesity in the offspring, via lifestyle interventions targeting behaviours that lead to altered nutrient partitioning and fetal overgrowth [10,13].

The effects of antenatal interventions on offspring outcome are difficult to establish. Birth weight is the most easily measured outcome assessing the impact of the intrauterine environment on fetal growth, [11] and we hypothesize that a reduction in fetal over-nutrition will be reflected as lower birth weight in offspring of overweight and obese mothers. The association between birth weight and adult weight suggests that there are enduring effects on later obesity risk [11], and we further postulate that reduction in birth weight will exert a protective effect on later obesity risk.

Antenatal exercise may play a positive role on both maternal and offspring health [15,16], and may be especially beneficial to the offspring of overweight and obese women [17]. While evidence on the effects of antenatal exercise on offspring outcomes (including birth weight) is inconsistent, there is no evidence of any adverse impact from moderate-intensity exercise [15,18]. Furthermore, the timing of antenatal exercise may be an important confounding factor leading to the varying effects of antenatal exercise on birth weight [17]. There is evidence that antenatal exercise in the second half of gestation can reduce fetal over-nutrition, birth weight and adiposity in the offspring, and protect against future obesity. Baraket et al. found that resistance exercise during the second and third trimesters of pregnancy attenuated the effect of increased maternal weight on offspring birth weight [19]. Clapp demonstrated that continuation of regular moderate- to vigorous-intensity antenatal exercise reduced offspring birth weight and subcutaneous fat mass at birth, with these potentially beneficial effects persisting up to 5 years of age [20], and that the maintenance of a high volume of exercise during mid and late pregnancy led to a reduction in fetal fat mass [21]. In a recent randomised controlled trial, we showed that non-weight-bearing aerobic exercise during the second half of pregnancy in non-obese women led to a significant reduction in offspring birth weight and BMI, in association with a reduction in metabolic markers of fetal nutrition [22].

Despite many studies investigating the effects of exercise during pregnancy, the effects of antenatal exercise in overweight and obese women on both short- and long-term health of the offspring are largely unknown. A recent systematic review of randomised controlled trials of antenatal exercise in overweight or obese women indicates that antenatal exercise may be beneficial in limiting gestational weight gain, but there is a lack of high-quality research evidence to assess the impact on offspring health [23]. Thus, the main aims of this trial are to establish if regular moderate-intensity, non-weight bearing exercise during the second half of pregnancy in overweight and obese women will reduce offspring birth weight and neonatal adiposity, and lead to long-term beneficial changes such as reduced obesity risk in the offspring. Secondly, we will explore the effects of antenatal exercise on fetal growth and body composition, maternal pregnancy and delivery outcomes, and maternal and cord blood metabolic and inflammatory markers. We will collect data on potential confounding/contributory factors including maternal weight gain, dietary intake, overall physical activity, physical fitness and quality of life indices during pregnancy.

## Methods/Design

The IMPROVE (Improving Maternal and Progeny Risks of Obesity Via Exercise) study is a two-arm parallel randomised controlled clinical trial being conducted in Auckland, New Zealand. This study is approved by the Health and Disability Ethics Committee (HDEC) (Ministry of Health, New Zealand; 12/NTB/24), and is registered with the Australian New Zealand Clinical Trials Registry (ACTRN 12612000932864). Locality approval has been obtained from the research offices at Auckland, Counties Manukau, and Waitemata District Health Boards, as well as relevant Māori research committees within the Auckland region.

### Participants

Pregnant women aged 18 to 40 years with a BMI ≥25 kg/m^2^ who are carrying a singleton fetus less than 20 weeks of gestation, with gestation confirmed by a dating scan early in pregnancy, and who are resident in the Auckland region are eligible for the trial. Exclusion criteria include ongoing smoking during the current pregnancy or contra-indications to aerobic exercise in pregnancy as stated by the American College of Obstetricians and Gynaecologists [24].

### Recruitment

The Auckland region has a population of approximately 1.4 million and approximately 21,000 births per year. Potential participants are idenified via maternity caregivers, posters and brochures in health care and community settings, and newspaper advertisements. All potential participants are supplied with detailed study information, and undergo an eligibility assessment. Eligible and willing participants are registered in the trial before completion of 20 weeks of gestation, and written informed consent obtained at the baseline visit. Target rate of recruitment is 8 participants per month, and recruitment is currently ongoing.

### Randomisation

After completion of baseline assessments, participants are randomised in a 1:1 ratio to either intervention or control group, using stratified blocked randomisation with variable block sizes of 2 and 4. The two stratification factors considered are parity (nulliparous/parous) and ethnicity (Maori/Pacific/New Zealand European or other).

Computer generated randomisation sequences have been provided by a biostatistician with no clinical involvement in the trial, and are stored securely in a password-protected computer. Once a participant is registered to the trial by the recruitment coordinator, a sequential study number and randomisation number is assigned to each participant. The recruitment coordinator does not have access to the randomisation tables. Group allocation is concealed until completion of baseline assessments.

### Intervention

The intervention group follow a regular structured exercise regime between 20 to 36 weeks of gestation, similar in nature to a previous antenatal exercise intervention conducted in non-obese women at our research institute [22]. The control group do not undergo any antenatal exercise intervention. Due to the nature of the intervention, participants are un-blinded to group assignment, following completion of baseline assessments.

The exercise regime consists of home-based stationary cycling. Magnetic exercycles (Sportop NB600/NB800) and heart rate monitors (Polar S625X/Polar RS800) are provided to each participant in the exercise group at 20 weeks of gestation. Exercise intensity is prescribed using target heart rate zones developed and validated specifically for overweight and obese pregnant women [25]. Participants receive instructions on equipment use and a written exercise prescription with weekly exercise targets. The 16-week exercise regime consists of 67 exercise sessions, comprising approximately 1500 minutes of moderate-intensity exercise. The frequency and duration of prescribed exercise varies over the intervention period. Participants are instructed to wear their heart rate monitors to record every prescribed exercise session they complete during the intervention period. This information is downloaded from each heart rate monitor to a computer at the end of the intervention period utilising Polar Precision Performance software (ProTrainer 5), so that compliance to prescribed exercise sessions can be assessed.

The intervention commences with 3 exercise sessions per week and increases to 5 sessions per week. All exercise sessions commence with a 5-minute warm-up on the stationary cycle at low intensity, maintaining heart rate below target rate, and concludes with a similar 5-minute cool down.The duration of moderate-intensity exercise at prescribed heart rate increases gradually from 15 minutes to 30 minutes per session and gradually decreases again from 33 weeks of gestation while maintaining the frequency at 5 sessions per week. A qualified exercise physiologist maintains regular contact with the exercising participants via phone, email, and home visits. To increase compliance with exercise, if cycling becomes unacceptable or uncomfortable with advancing pregnancy, participants are encouraged to utilise an alternative forms of exercise such as brisk walking, while maintaining same intensity, frequency, and duration of exercise.

Over the duration of the intervention period, both groups are free to continue their normal exercise/physical activity routines and receive standard antenatal care, and undergo similar study assessments. In addition, there are no dietary restrictions. Exercise participants developing obstetric contra-indications to exercise [24] will discontinue the exercise intervention, but be included in the intention to treat analysis.

### Primary outcome

Primary outcome is offspring birth weight, measured in grams (g) and standardized z-score (i.e. corrected for gestational age and gender) [26]. As there are significant differences in birth weight between major ethnic groups in New Zealand, birth weight will also be compared by customised centiles adjusting for maternal height, weight, parity, ethnic origin, gestational age at birth, and gender of the baby [27].

### Secondary outcomes

Offspring outcomes include neonatal anthropometry and body composition, fetal growth measures, cord blood metabolic markers and newborn complications including rates of large-for-gestational-age (birth weight >90^th^ centile) and small-for-gestational-age (birth weight <10^th^ centile) babies. Maternal outcomes include weight gain, aerobic fitness, quality of life, metabolic markers, post-partum weight and body composition, as well as pregnancy and delivery outcomes including timing and mode of delivery and length of hospital stay.

### Other aims and outcomes

This study also aims to establish a prospective mother-offspring cohort for follow-up to determine the long-term effects of antenatal exercise on anthropometry, body composition, and obesity risk.

### Clinical assessments/Data collection

Each participant visits the Maurice and Agnes Paykel Clinical Research Unit (Liggins Institute, Auckland) for three study assessments. The first visit for the baseline assessment occurs at 19 ± 1 weeks of gestation and the second visit at 36 ± 1 weeks of gestation. During the first visit, participants complete consent forms and fill in questionnaires on demographic, medical and obstetric history. The other procedures are similar for both first and second visits, where each participant’s weight, height, resting heart rate, resting blood pressure, HbA1c, and baseline aerobic capacity are assessed by a single investigator. Blood samples are collected and stored for metabolic testing. Participants complete a quality of life assessment questionnaire and the physical activity questionnaire and 3-day diet records are collected. At the second visit participants receive a pack with instructions for collection of cord blood by delivery staff.

During the intervention period, participants in both groups undergo regular ultrasound scans for research purposes at 4-week intervals starting from 24 ± 2 weeks of gestation. Participants are requested to maintain daily records of any leisure time physical activity/exercise they engage in between 20 weeks of gestation up to delivery on exercise diaries provided by the study. Participants also complete 3-day food intake records and a physical activity questionnaire at baseline prior to randomisation and midway though the intervention at a time when exercise prescription is maximal (32 weeks of gestation), to assess any group difference in these measures.

The final study visit occurs at 14 ± 2 days after delivery. Maternal weight and neonatal anthropometry (weight, length and occipito-frontal head circumference) are recorded, and both mother and baby have body composition assessed by DXA scans. Pregnancy, delivery and newborn data (including feeding history) are obtained from the participants and hospital records. Study exercise diaries and heart rate monitors are also collected at this point.

### Antenatal measures - maternal

Maternal body weight is measured to the nearest 0.1 kg using calibrated electronic scales (Tanita, USA). Maternal height is measured to the nearest millimetre using a fixed wall Harpenden Stadiometer (Holtain Ltd., Crymych, UK). Resting heart rate and resting blood pressure are measured using standard protocols [28].

Aerobic fitness testing is carried out using a sub-maximal graded exercise test on an electronically-braked cycle ergometer (Schiller, Baar, Switzerland), with simultaneous breath-by-breath measurement of expired and inspired O_2_ and CO_2_ gas volumes (ParvoMedics TrueOne 2400 Metabolic Measurement System, Parvomedics, Sandy, Utah, USA) to a target heart rate of 150 beats per minute, as previously described [22]. The test begins with a workload of 30 W and increases by 10 W every minute until a target heart rate of 150 beats per minute is reached. Participants are instructed to maintain a constant cycling speed at 60 rpm throughout the test. Parameters of interest include time taken to reach the peak heart rate of 150 bpm, workload (W) when reaching peak heart rate, and peak VO_2_.

Maternal quality of life is measured using a self-administered questionnaire, the WHO Quality of Life-BREF (WHOQOL-BREF) version, which consists of a total of 26 questions in 4 domains [29]. Maternal physical activity levels are measured using the Pregnancy Physical Activity Questionnaire (PPAQ). The PPAQ is self-administered and asks respondents to report the time spent participating in 32 activities, including household/caregiving, occupational, sports/exercise, transportation, and sedentary activities [30]. The total weekly energy expenditure in MET-h/week is measured. Dietary data from 3-day food records completed at baseline and mid intervention are analysed using Foodworks 7 Professional edition (Xyris Software, Australia). Data are entered into the database by a single investigator blinded to group allocation, and analysed for total energy intake as well as% energy from protein, fat, and carbohydrate.

### Antenatal measures - offspring

Measurement of fetal growth are performed using standard obstetric ultrasound techniques by a single investigator, blinded to group allocation, at a single medical institution in Auckland (Middlemore Hospital, Counties Manukau District Health Board). All scans are performed utilising a single Philips IU22 ultrasound scanner (Philips Ultrasound, Bothell, USA) with 3D capability. The ultrasound transducer adopted is the Philips curvilinear array (C5-1, Philips Ultrasound, Bothell, USA) while the 3D transducer is a mechanical curved-array 3D abdominal ultrasonic transducer (3D 6–2 Philips Ultrasound).

Standard growth measurements including biparietal diameter (BPD), head circumference (HC), humerus diaphyseal length (HDL), abdominal circumference (AC), and femur diaphyseal length (FL) are performed according to the protocols published by the Australasian Society for Ultrasound in Medicine [31]. At each examination, all measurements are obtained three times from three separately generated ultrasound images, and then averaged. The standard measurements are used to calculate estimated fetal weight (EFW) by the Hadlock 4 equation [32]. Scans are recorded electronically for later analysis of other measures of fetal growth and fat deposition. Soft tissue growth is assessed by measurements taken from the 3D volume dataset including thigh circumference (ThC), partial thigh volume (TVol), arm circumference, (ACirc) and partial arm volume (AVol) as described by Lee [33]. The 3D volume measurements are obtained by averaging three measurements from three manipulations of the one data set offline. Volume analysis is done offline using Philips QLAB™ software version 6. Measurements of fetal fat mass are calculated by taking the total cross-sectional limb area and subtracting the central lean area, as previously described [34].

Between-group comparisons of fetal growth will include measures of BPD, HC, AC, HDL, FL and EFW at 24, 28, 32, and 36 weeks of gestation. Between-group comparisons of fetal body composition will include measures of ThC, TVol, ACir, AVol and estimated fat mass at 28, 32, and 36 weeks of gestation.

### Postnatal measures

Newborn weight, length, and head circumference measured at birth are obtained from medical records. Data on Apgar scores at 1 and 5 minutes after birth, neonatal hypoglycaemia needing intravenous treatment, neonatal hyperbilirubinaemia requiring treatment, presence of birth injuries, congenital anomalies, admission to neonatal intensive care or special care units, and length of hospital stay following delivery are also collected. Neonatal anthropometry (weight, length, and head circumference) are measured by a single investigator at the Maurice and Agnes Paykel Clinical Research Unit at 14 ± 2 days after delivery. Neonatal weight is measured to the nearest 10 g using electronic scales (Tanita 1583, Tanita Corp). Neonatal length is measured using a Holtain neonatometer. Occipito-frontal head circumference is obtained to the nearest millimetre using a flexible non-stretchable tape. BMI is calculated as weight/height^2^ (kilograms/metre^2^). Ponderal index is calculated as weight in grams × 100 divided by the cube of length in centimetres.

Maternal and neonatal body composition and bone density are evaluated at 14 ± 2 days after delivery by whole-body dual-energy absorptiometry (DXA, Lunar Prodigy 2000, General Electric, Maddison, Wisconsin, USA), by a single investigator at the Maurice and Agnes Paykel Clinical Research Unit. All scans are analysed by the same operator, utilising Encore 2007 software v.11.40.004 including paediatric software packages (GE Corp., Madison, WI, USA). Quality control checks are performed on a daily basis prior to scanning, using a standard block provided by the manufacturer.

### Metabolic markers

Maternal blood samples collected at baseline and at the end of intervention are processed and stored at the Liggins institute. Venous cord blood is collected at delivery by the lead maternity carer, who is instructed beforehand on the procedure. Cord blood samples are processed within 3 hours of collection and stored at -80°C at hospital laboratories. We plan to analyse maternal and cord blood samples at the end of the study. Parameters that will be assessed include: insulin-like growth factors I (IGF-I), IGF-II, insulin-like growth factor binding protein 1 (IGFBP-1), IGFBP-3, leptin, adiponectin, interleukin 6 (IL-6), tumour necrosis factor α (TNF α), insulin, glucose, highly sensitive C-reactive protein (CRP), triglycerides, total cholesterol, high-density lipoprotein cholesterol (HDL-C), low-density lipoprotein cholesterol (LDL-C) and free fatty acids. Sex hormone-binding globulin (SHBG) will also be measured in maternal samples as a surrogate measure of insulin resistance. Samples will be preserved for future metabolomics studies.

### Statistical considerations

#### ***Sample size***

Based on a previous exercise intervention performed by our research group in Auckland using similar methodology [22] and using birth weight as the primary outcome, a total sample size of 100 women (n = 50 per group) will provide 80% power at 5% level of significance (two-sided) to detect a group difference of 250 g or more, assuming a standard deviation of 430 g [22].

#### ***Data analyses***

Statistical analyses will be performed using SAS version 9.3 (SAS Institute Inc. Cary NC). All statistical tests will be two-tailed and a 5% significance level maintained throughout the analyses. Study data are entered into an Excel database, and then imported into SAS for final analysis.

#### ***Baseline characteristics***

Baseline characteristics will be summarised using descriptive statistics. Continuous variables will be described as numbers of observed and missing values, mean, standard deviation, median, minimum and maximum. Categorical variables will be described as frequencies and percentages. Results will be presented for each of the two treatment arms as well as overall. Since any differences between randomised groups at baseline could only have occurred by chance, no formal significance tests will be conducted.

#### ***Treatment effects***

Treatment evaluation will be performed on the principle of intention to treat (ITT), using data collected from all randomised participants. Analysis of covariance (ANCOVA) regression models will be used to evaluate the main treatment effect on the primary outcome between the two treatment groups, adjusting for parity and maternal ethnicity (i.e. stratification factors). A similar approach will be used for other continuous secondary outcomes, with the baseline outcome (if measured) added to the model as a confounding variable. Model-adjusted means and their difference between two groups will be estimated and tested. Logistic regression model will be used for the analysis of a binary outcome (i.e. yes or no), with associated odds ratio and 95% confidence interval. In addition to the intention to treat analysis, a per protocol (PP) analysis will also be undertaken on those participants who have no major protocol violations.

Reporting will adhere to the CONSORT guidelines for reporting parallel group randomised trials [35,36].

## Discussion

This randomized controlled trial has several unique characteristics. First, it utilises a non-weight bearing home-based antenatal exercise intervention, previously applied to non-obese women [22]. This will enable direct comparison of outcomes of a similar exercise intervention in obese and lean women. Secondly, the main focus of this antenatal exercise trial is on offspring health, with longitudinal assessments of fetal growth and body composition during the intervention period, as well as measures of newborn anthropometry and adiposity. While a number of randomised controlled trials of prescribed antenatal exercise have been conducted over the last three decades, their primary focus has been on maternal health and there is a lack of robust evidence on the effects of antenatal exercise on offspring health (18, 23). Furthermore, while there is some evidence of positive long-term effects of antenatal exercise for offspring of lean mothers (20), long-term effects on offspring of overweight or obese mothers appear to have received little attention. Therefore, this trial also aims to establish a cohort of overweight /obese mothers and offspring with well-documented antenatal and perinatal data to be followed up to determine the long-term effects of antenatal exercise on offspring and maternal health. As a result, this study addresses the lack of prospective robust data on the effects of antenatal exercise on short- and long-term outcomes of mothers and their offspring (43).

Another feature of this trial is the use of methods to enhance and monitor compliance to prescribed exercise. Many antenatal exercise interventions, especially those involving overweight and obese women have encountered problems with compliance [37,38]. This may be partly attributed to the general tendency for physical activity levels to decline during pregnancy [39,40], especially in mothers who are obese [41,42], However, many previous exercise studies have also failed to report adequately on compliance with interventions [18]. Previous studies have suggested that home-based exercise interventions can lead to increased compliance, as they enable exercise to be undertaken in a comfortable and familiar environment [43]. Therefore, a home-based intervention was chosen, to enhance feasibility of regular exercise, and improve compliance to the exercise protocol. Previous home-based stationary cycling interventions in non-obese [22] and obese [43] pregnant women have reported high acceptance and good compliance levels. Furthermore, during pregnancy, non-weight bearing exercises may be better tolerated, minimizing joint and musculoskeletal stress [44], and stationary cycling can be especially suitable for overweight or obese women, who are likely to continue to gain extra weight with advancing pregnancy. In addition, exercise prescription in our study has been modified to suit sedentary overweight and obese pregnant women, with gradual progressive increase in frequency and duration in keeping with recommendations [44], while maintaining moderate intensity based on target heart rates specific for this group [25].

While this study has been designed to optimise compliance, it remains essential to have objective assessment of compliance. This has been absent from many exercise intervention trials, making assessment of benefits problematic. In this study, we are using downloadable heart rate monitors. Not only does this provide evidence of exercise intensity, but also evidence of compliance that can be used in outcome analysis.

In summary, the proposed study will provide a platform to objectively assess a non-weight bearing exercise regime on overweight and obese women pregnant women, provide quantitative outcome data on them and their babies, and lead to an offspring cohort that can be followed throughout childhood.

## Abbreviations

AC: Abdominal circumference; ACirc: Arm circumference; ANZCTR: Australian New Zealand Clinical Trials Registry; AVol: Partial arm volume; BMI: Body mass index; BPD: Biparietal diameter; CO2: Carbon dioxide; CRP: C-reactive protein; DXA: Dual-energy absorptiometry; FL: Femur diaphyseal length; HbA1c: Glycosylated haemoglobin; HC: Head circumference; HDEC: Health and Disability Ethics Committee; HDL-C: High-density lipoprotein cholesterol; HDL: Humerus diaphyseal length; IGF: Insulin-like growth factor; IGFBP: Insulin-like growth factor binding protein; IL: Interleukin; LDL-C: Low-density lipoprotein cholesterol; MET: Metabolic equivalent; PPAQ: Pregnancy Physical Activity Questionnaire; SHBG: Sex hormone-binding globulin; ThC: Thigh circumference; TNF: Tumor necrosis factor; TVol: Partial thigh volume; VO2: Oxygen consumption.

## Competing interests

The authors declare that they have no competing interest.

## Authors’ contributions

SNS drafted the manuscript, was involved in study conception and design, coordinates the trial, and performs data acquisition and interpretation. GKP participated in design of the study and is involved in fetal growth data acquisition and analysis. LMEM contributed to design of the study and is involved in pregnancy and obstetric data analysis. AE contributed to conception and design of the study including cultural adaptation to suit the Pacific Island community. YJ contributed to study design and is involved in statistical analysis. SG participated in designing the exercise intervention and fitness assessments. ROR assists with dietary data analysis. SC assists with recruitment and study coordination. GP helped design and coordinate the exercise intervention. WSC contributed to study conception and design. PLH was involved with conception and design of the trial and supervises the research team. SNS, GKP, LMEM, YJ, WSC and PLH have been involved in revising the draft critically for important intellectual content. All authors have given final approval of the version to be published.

## Pre-publication history

The pre-publication history for this paper can be accessed here:

http://www.biomedcentral.com/1471-2393/14/148/prepub
